# Seasonal Climate Drivers and Immunohematological Profiles of Malaria in Rohingya Refugee Camps, Bangladesh: A 4‐Year Repeated Cross‐Sectional Study

**DOI:** 10.1155/japr/6044433

**Published:** 2026-04-27

**Authors:** Ashekul Islam, Maria Mehjabin Akhi, Akram Hossain, Pulak Kanti Palit, Nafsoon Rahman, Sunanda Baidya, Tabassuma Marzia Prome, Mishu Rahman, Afroza Sultana, Mohammed Mehadi Hassan Chowdhury, Md. Shah Alam

**Affiliations:** ^1^ Department of Biochemistry and Molecular Biology, Laboratory of Vector-Borne and Infectious Diseases, Mawlana Bhashani Science and Technology University, Tangail, Bangladesh, mbstu.ac.bd; ^2^ Department of Statistics, Mawlana Bhashani Science and Technology University, Tangail, Bangladesh, mbstu.ac.bd; ^3^ Department of Medicine, Chittagong Medical College Hospital, Chattogram, Bangladesh; ^4^ Department of Biochemistry and Molecular Biology, Jagannath University, Dhaka, Bangladesh, jagannathuniversity.org; ^5^ Department of Biochemistry and Molecular Biology, Laboratory of Immune Signaling, University of Chittagong, Chittagong, Bangladesh, cu.ac.bd; ^6^ Infectious Diseases Division, International Centre for Diarrhoeal Disease Research, Bangladesh (icddr b), Dhaka, Bangladesh, icddrb.org; ^7^ Department of Zoology, Laboratory of Entomology, Jagannath University, Dhaka, Bangladesh, jagannathuniversity.org; ^8^ Department of Microbiology, Noakhali Science and Technology University, Noakhali, Bangladesh, nstu.edu.bd; ^9^ Department of Parasitology and Pathology, Patuakhali Science and Technology University, Barishal, Bangladesh, pstu.ac.bd

**Keywords:** climate variability, hematological biomarkers, *Plasmodium falciparum*, Rohingya refugees, serological surveillance

## Abstract

**Background:**

Malaria remains a critical public health challenge in southeastern Bangladesh, particularly in Cox′s Bazar, where the mass displacement of over one million Rohingya refugees since 2017 has heightened transmission risks. The interplay of ecological fragility, overcrowded living conditions, and climatic variability underscores the need for a deeper understanding of malaria epidemiology in this high‐risk setting.

**Methods:**

A 4‐year repeated cross‐sectional study was conducted (2021–2024) in Camp No. 26, Teknaf, Cox′s Bazar. A total of 582 participants were enrolled, comprising 486 individuals from the malaria‐endemic refugee camp and 96 healthy controls from a nonendemic region. All were screened for *Plasmodium falciparum* and *Plasmodium vivax* using rapid diagnostic tests (RDTs), with microscopy performed to confirm all RDT‐positive cases and a subset of RDT‐negative samples. Meteorological data (temperature, rainfall, and humidity) were obtained from regional weather stations. Serological profiling assessed total anti‐*Plasmodium* antibodies, while hematological parameters including hemoglobin concentration, RBC and platelet counts, and ESR were measured. Correlation and regression analyses were employed to identify climatic predictors of malaria incidence and their associations with immunohematological changes.

**Results:**

Out of 582 individuals, 345 malaria cases were confirmed. Peak transmission occurred during the monsoon season (June–September), particularly in August. *P. falciparum* infections showed earlier and sharper peaks compared to *P. vivax*. Relative humidity demonstrated the strongest correlation with incidence (*r* = 0.724–0.77), followed by rainfall and temperature. Antibody titers were significantly higher in *P. falciparum*–positive individuals. Infected participants exhibited anemia, thrombocytopenia, and elevated ESR, with more pronounced alterations in *P. falciparum* cases.

**Conclusion:**

This study highlights the seasonal and species‐specific nature of malaria transmission in Rohingya refugee camps, driven predominantly by climatic variables, particularly humidity. The observed serological and hematological alterations underscore their potential as biomarkers for surveillance. These findings advocate for climate‐sensitive, species‐specific malaria control strategies tailored to displaced populations.

## 1. Introduction

Malaria, a mosquito‐borne disease transmitted by female *Anopheles* mosquitoes, remains a substantial public health burden, with an estimated 263 million cases and 597,000 deaths worldwide in 2023 [1]. It is caused by parasites of the genus *Plasmodium*, which pose a significant threat, with nearly half of the world′s population at risk. Children and pregnant women are particularly vulnerable due to their developing or compromised immune system, and in 2023, it was estimated that around three‐quarters of global malaria deaths occurred in children under 5 [2]. In Bangladesh, malaria persists as a public health issue, particularly in 13 endemic districts bordering India and Myanmar that account for about 98% of reported cases [3]; despite a 93% reduction in incidence between 2008 and 2020, around 18 million people remain at risk due to climatic and ecological factors driving geographic disparities in transmission, underscoring the need for context‐specific strategies to achieve elimination by 2030 [4].

*Anopheles* mosquitoes require a moderate temperature ranging from 18°C to 32°C and a relative humidity of at least 60% for optimal growth and parasite transmission [5]. Minor temperature increases (e.g., 1°C within 18°C–26°C) can prolong mosquito lifespan, enhancing transmission potential [6]. Rainfall also plays a critical role: While increased precipitation creates stagnant water that serves as breeding grounds for mosquitoes, excessive rainfall can flood these sites, leading to a decline in mosquito reproduction and malaria transmission [7]. Conversely, low humidity (< 60%) reduces mosquito survival and parasite development [8]. These findings highlight the substantial influence of climatic conditions on malaria incidence. Human‐driven environmental changes, including deforestation and population displacement, further influence local transmission dynamics [9]. Moreover, coastal climates, in particular, exhibit less variability than inland areas due to the thermal inertia of nearby water bodies. As oceans and seas heat and cool more slowly than land, temperature and humidity in coastal regions remain relatively stable [10]. Similarly, coastal regions routinely experience convective precipitation and are typically more humid. This unique climatic behavior becomes particularly relevant when assessing malaria transmission in coastal regions like Cox′s Bazar.

Cox′s Bazar, situated in the southeastern coastal belt of Bangladesh, is not only a prominent tourist destination but also a critical hotspot for malaria transmission, driven by its unique geographic, climatic, and demographic conditions. Although it shares a tropical monsoon climate with much of the country, its coastal geography amplifies rainfall, humidity, and temperature extremes, particularly during the southwest monsoon, creating optimal conditions for *Anopheles* mosquito breeding and malaria transmission. The proximity of Cox′s Bazar to the Myanmar border further complicates malaria control efforts. Cross‐border ecological continuity and population movement, especially from Myanmar′s Rakhine and Chin States, areas with intense malaria transmission, compromise surveillance and containment strategies [11]. Sinha et al. confirm that Cox′s Bazar exhibits seasonal malaria transmission, with *Plasmodium falciparum* accounting for over 70% of infections [12]. However, *Plasmodium vivax* and *Plasmodium malariae* have also been detected, underscoring species diversity and potential hypnozoite‐related relapses among both host communities and displaced populations [13].

Following the mass displacement of over one million Rohingya refugees since 2017, with further influxes in 2023 due to ongoing conflict and instability [14], Cox′s Bazar now hosts some of the world′s most densely populated refugee camps. These settlements face challenges such as poor sanitation, limited healthcare, and inadequate vector control that elevate malaria vulnerability, particularly among immunologically naïve or malnourished individuals. Seroepidemiological surveys indicate that malaria antibody levels and infection rates are significantly higher among Rohingya children and adults compared to local residents, suggesting ongoing transmission [11]. Despite national progress in malaria control, supported by the Global Fund and local nongovernmental organizations (NGOs), these combined pressures of climate variability and forced migration pose a serious threat to sustained elimination efforts. Thus, there is an urgent need for evidence‐based research to understand how ecological stressors and human displacement interact to influence malaria dynamics in this high‐risk zone. Accordingly, this study is aimed at assessing the impact of seasonal climatic variations on malaria incidence in Cox′s Bazar between 2021 and 2024. It also seeks to evaluate how refugee burden affects transmission patterns and control efforts, focusing on *Plasmodium* species profiling, immunohematological markers, and longitudinal infection surveillance. By correlating meteorological variables such as rainfall, temperature, and humidity with infection rates, the study will contribute to predictive modeling, adaptive vector control, and cross‐border health policy development in complex humanitarian settings.

## 2. Materials and Methods

### 2.1. Study Area, Subjects, and Blood Sample Collection

This study was conducted in Camp No. 26, located in Teknaf, Cox′s Bazar, Bangladesh (geographic coordinates: 20.951036° N, 92.254366° E), an area with a persistent malaria burden, particularly among the Rohingya refugee population, an ethnic minority group residing in the region. Malaria transmission in this setting is perennial with distinct seasonality, with the majority of cases occurring during the rainy season, typically spanning from May to mid‐October [15]. Between 2021 and 2024, a total of 486 individuals from the refugee camp (considered an endemic region, EnR) and 96 healthy individuals (malaria‐free reference population rather than a matched control group) from Santosh, Tangail (a nonendemic region [non‐EnR]), were enrolled in the study. This region was selected due to the absence of malaria transmission during the study period and its epidemiological stability. Given the restricted mobility of Rohingya refugees, there is no population overlap, ensuring independence between groups. Participants presenting with malaria‐like symptoms were selected using a systematic random sampling approach within each survey round, stratified by age and sex to ensure representativeness. All participants were initially screened using a malaria rapid diagnostic test (RDT) kit for *P. falciparum* and *P. vivax* (Synthgene, Xianlin, China), and phase contrast microscopy (Olympus BX40, Japan) was performed as a confirmatory method on all RDT‐positive samples and a subset of RDT‐negative samples. Relevant clinical and demographic data including date of diagnosis, age, and sex were recorded for each participant. For serological analyses, 2–3 mL of peripheral blood were aseptically collected via venipuncture into EDTA‐coated vacutainer tubes (BD Vacutainer, New Jersey 07417‐1885, United States). Samples were immediately placed on ice and transported to the nearby field laboratory on the same day for further processing. Written informed consent was obtained from all participants or their legal guardians prior to enrolment; in the case of minors, assent was also obtained. Trained medical personnel conducted structured interviews using a pretested questionnaire to collect demographic and epidemiological information. The study protocol was reviewed and approved by the Institutional Review Board (IRB) of Mawlana Bhashani Science and Technology University, Tangail, Bangladesh.

### 2.2. Meteorological Data

Meteorological data, including daily temperature, rainfall, and relative humidity, were obtained from the regional meteorological station located in Cox′s Bazar, Bangladesh, for the duration of the study period (source: Bangladesh Meteorological Department [BMD]; http://www.bmd.gov.bd/). However, metadata pertaining to the instrumentation (e.g., sensor type and calibration standards), spatial representativeness, measurement uncertainty, or station‐specific variograms for climatic parameters were not disclosed by the data provider. Despite these constraints, data from BMD stations are widely used in climate–health studies in Bangladesh due to their accessibility and regional relevance.

### 2.3. Antibody Profiling

A total of 582 serum samples collected from all study participants were tested for total anti‐*Plasmodium* spp. antibodies using the Malaria EIA kit (Newmarket Laboratories, Bio‐Rad, United States). This ELISA‐based assay is designed to detect total antimalarial antibodies (IgG, IgM, and IgA) against *Plasmodium* species using specific recombinant antigens. The procedure was carried out following the manufacturer′s instructions. In brief, 50 *μ*L of undiluted serum from each participant was added to the appropriate wells of a microtiter plate. Each assay included positive controls (run in duplicate) and negative controls (run in triplicate). After gently shaking the plate for 30 s, it was covered and incubated at 37°C for 30 min. Following incubation, the wells were washed five times using the supplied wash buffer. Subsequently, 50 *μ*L of horseradish peroxidase (HRP)–conjugated secondary antibody, prepared according to the instructions, was added to each well, followed by a second 30‐min incubation at 37°C. The wells were again washed five times, and 50 *μ*L of substrate solution was added. The plate was then incubated in the dark for 30 min. The reaction was stopped by adding 50 *μ*L of 0.5 M sulfuric acid to each well, and absorbance was measured at 450 nm using a microplate reader. The antibody index for each sample was calculated by dividing the optical density (OD) of the sample by the cutoff value. The cutoff was determined as the mean OD of the negative controls plus 0.100, in accordance with the kit protocol.

### 2.4. Hematological Assays

Hemoglobin concentration was determined using the cyanmethemoglobin method, a standard colorimetric assay [16]. In this procedure, 20 *μ*L of thoroughly mixed EDTA‐anticoagulated blood was added to 5 mL of Drabkin′s reagent (containing potassium ferricyanide and potassium cyanide in phosphate buffer, pH 7.0). The mixture was incubated at room temperature to allow complete conversion of hemoglobin to cyanmethemoglobin. Absorbance was measured at 540 nm using a UV‐visible spectrophotometer (e.g., Shimadzu UV‐1800), and concentrations were derived by comparing the readings to a standard curve generated using commercially available cyanmethemoglobin standards (Sigma‐Aldrich, United States).

Red blood cell (RBC) and platelet counts were performed manually using a Neubauer hemocytometer following the protocols described. For RBC count, blood was diluted at a 1:200 ratio with Hayem′s solution; for platelet count, a 1:20 dilution was prepared using 1% ammonium oxalate solution. The diluted samples were charged into the hemocytometer chambers and allowed to settle for 2–3 min. Cell counting was performed under a light microscope (Olympus CX23, Japan) using a 40× objective. The cell counts were calculated using standard formulas and expressed as ×10^6^/*μ*L for RBCs and ×10^3^/*μ*L for platelets.

Erythrocyte sedimentation rate (ESR) was assessed using the Westergren method [17], as recommended by the International Council for Standardization in Hematology (ICSH, 1993). Citrated blood was drawn into Westergren tubes up to the 200 mm mark and placed vertically in ESR racks at room temperature (20°C–25°C), away from direct light and vibrations. After 60 min, the length of the plasma column formed above the sedimented RBCs was recorded in millimeters and reported as ESR (millimeters per hour).

### 2.5. Data Analysis

Statistical analyses were performed to examine the relationship between weather variables, malaria transmission, and antibody dynamics. Descriptive statistics were used to summarize the weather data, malaria case data, and antibody levels. Correlation analysis was conducted to assess the association between weather variables and malaria transmission. Data analysis was carried out using either SPSS (V 2000), GraphPad (V 10.4.2) (United States), or R (V 4.3.4), and significance levels were set at *p* < 0.05. To account for multiple comparisons in hematological, clinical, and serological variables, *p* values were adjusted using the Holm–Bonferroni method, and adjusted *p* values < 0.05 were considered statistically significant. Statistical analyses were conducted to investigate the associations between meteorological variables, malaria transmission trends, and antibody response dynamics. Descriptive statistics were used to summarize key features of the climatic data (temperature, rainfall, and humidity), malaria incidence rates, and serological measurements. Pearson or Spearman correlation analyses, as appropriate based on data distribution, were applied to evaluate the strength and direction of associations between weather parameters and malaria cases. Lagged correlation analysis was performed by shifting meteorological variables by 0, 1, and 2 months relative to infection rates to account for potential delayed effects of climatic factors on malaria transmission. Correlation coefficients (*r*) and corresponding *p* values were calculated for each lag period to assess both the magnitude and statistical significance of associations.

## 3. Results

A total of 582 individuals were enrolled in this study, including 96 participants from a non‐EnR (nonendemic region healthy control [non‐EnR HC]), 141 healthy controls from the EnR who tested negative for malaria (endemic region healthy control [EnR HC]), 126 individuals infected with *P. vivax* (*P. vivax* antigen positive [PvAg+]), and 219 individuals infected with *P. falciparum* (*P. falciparum* antigen positive [PfAg+]) (Table 1). Demographic analysis showed no significant differences in age or sex distribution across the groups (*p* > 0.05), confirming baseline comparability. Clinically, individuals with malaria infection presented with significantly elevated body temperatures compared to both healthy control groups. The mean body temperature was 38.24°C ± 0.29°C in PvAg+ cases and 38.06°C ± 0.36°C in PfAg+ cases, whereas the nonendemic and endemic healthy controls showed mean temperatures of 36.71°C ± 0.32°C and 36.63°C ± 0.34°C, respectively (*p* ≤ 0.001). Parasitemia levels were comparable between *P. vivax* and *P. falciparum* infections (6773 ± 1867 vs. 6925 ± 2580 parasites/*μ*L), with no statistically significant difference observed (Table 1).

**Table 1:** Demographic, hematological, and serological characteristics of study participants.

Variable	Non‐EnR HC (*n* = 96)	EnR HC (*n* = 141)	PvAg+ (*n* = 126)	PfAg+ (*n* = 219)	*p* value (adjusted)
Age (years, mean ± SD)	34.94 ± 6.21	33.84 ± 7.14	35.28 ± 6.83	33.06 ± 5.62	n.s.
Body temperature (°C, mean ± SD)	36.71 ± 0.32	36.63 ± 0.34	38.24 ± 0.29	38.06 ± 0.36	p ≤0.001 ^∗^
Parasitemia (no./*μ*L)	—	—	6773 ± 1867	6925 ± 2580	n.s.
Hb (g/L)	13.97 ± 1.69	13.10 ± 1.64	8.35 ± 1.87	7.94 ± 1.74	p ≤0.001 ^∗^
ESR (mm/h)	11.47 ± 2.36	17.00 ± 4.93	39.13 ± 3.18	43.16 ± 3.12	p ≤0.001 ^∗^
RBC (millions/*μ*L)	4.661 ± 0.013	4.626 ± 0.030	4.113 ± 0.115	4.093 ± 0.058	p ≤0.001 ^∗^
Platelets (×10^3^/*μ*L)	272.0 ± 38.6	241.7 ± 36.7	88.6 ± 5.81	84.8 ± 3.91	p ≤0.001 ^∗^
Anti‐*Pv* antibodies	BDL	1.21 ± 0.08	2.08 ± 0.18	—	p ≤0.001 ^∗^
Anti‐*Pf* antibodies	BDL	1.44 ± 0.21	—	2.35 ± 0.21	p ≤0.001 ^∗^

Abbreviations: BDL, below detection level; n.s., not significant; *Pf*, *Plasmodium falciparum*; *Pv*, *Plasmodium vivax*.

^∗^ *p* values adjusted for multiple comparisons using the Holm–Bonferroni method.

### 3.1. Temporal Trends and Seasonal Dynamics of Malaria Transmission

A comprehensive analysis of malaria surveillance data collected between 2021 and 2024 in the Rohingya refugee Camp No. 26, Teknaf in Cox′s Bazar, Bangladesh, was undertaken to evaluate the prevalence and temporal dynamics of malaria transmission. Over the 4‐year surveillance period, malaria incidence exhibited clear seasonal trends, with consistently elevated case numbers during the monsoon season (June–September), reaching a peak in July and August. In contrast, malaria cases declined markedly during the cooler and drier months (November–March). These findings highlight a strong seasonal dependency of malaria transmission in this refugee setting and suggest a significant correlation between climatic conditions and malaria prevalence.

### 3.2. Monthly Distribution of Plasmodium Species Infections

**Figure 1:**
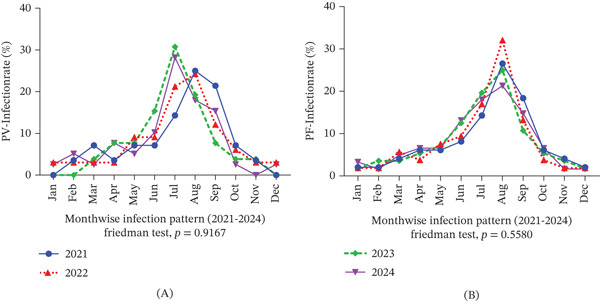
Monthly distribution of *Plasmodium vivax* and *Plasmodium falciparum* cases in the study camp (2021–2024). (A) *P. vivax* cases show a consistent seasonal peak during the monsoon months, with the highest incidence in July and August across all years. (B) *P. falciparum* cases peak sharply in August each year, indicating a stable seasonal transmission pattern, with elevated cases in adjacent months. Data are shown per individual participant: nonendemic healthy controls (*n* = 96), endemic healthy controls (*n* = 141), *P. vivax* positive (*n* = 126), and *P. falciparum* positive (*n* = 219). Percentages were calculated using the number of participants in each respective group as the denominator.

Species‐specific analysis revealed distinct temporal patterns in *P. vivax* and *P. falciparum* infections (Figure 1). *P. vivax* incidence peaked in August and September of 2021, July and August of 2022, and July of both 2023 and 2024. Meanwhile, *P. falciparum* demonstrated consistently high activity in August across all 4 years, with notable surges also occurring in September 2021 and adjacent months. These patterns suggest a reliable seasonal transmission cycle, with *P. falciparum* peaking slightly earlier and more sharply than *P. vivax*, marking August as a critical window for targeted interventions.

### 3.3. Climatic Drivers of Malaria Transmission

**Figure 2:**
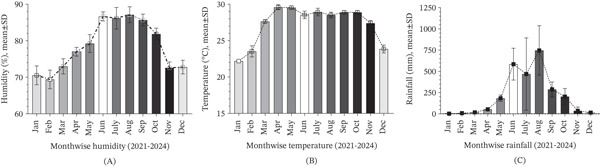
Monthly variation of climatic factors and malaria incidence in the study camp (2021–2024). (A) Relative humidity (%) and malaria cases exhibit a synchronized seasonal trend, with humidity rising from March and peaking in August, corresponding to increased transmission. (B) Average temperature (°C) shows a gradual rise from March, peaking in April–May and remaining elevated through October, aligning with heightened malaria incidence. (C) Monthly rainfall (millimeters) increases from April and peaks in August, coinciding with the highest malaria case counts, particularly *P. falciparum*, indicating a strong link between precipitation and transmission intensity.

Monthly meteorological analysis revealed a clear temporal association between environmental conditions and malaria incidence (Figure 2). Relative humidity increased progressively from March, peaking in August (mean ~87%), in close synchrony with malaria case surges. This correlation highlights humidity as a key factor enhancing mosquito survival and *Plasmodium* development. Temperature followed a bimodal rise, increasing from March and peaking in April–May, with sustained elevations through October. This thermal window aligned with the onset and persistence of malaria transmission, reinforcing its role in facilitating vector proliferation. Rainfall patterns revealed a steady rise from April, peaking in August, a period coinciding with peak *P. falciparum* cases. The synchronization between precipitation and malaria transmission underscores the contribution of monsoonal rainfall in creating optimal breeding habitats for *Anopheles* mosquitoes.

### 3.4. Correlation Between Meteorological Factors and Malaria Infection Rates

**Figure 3:**
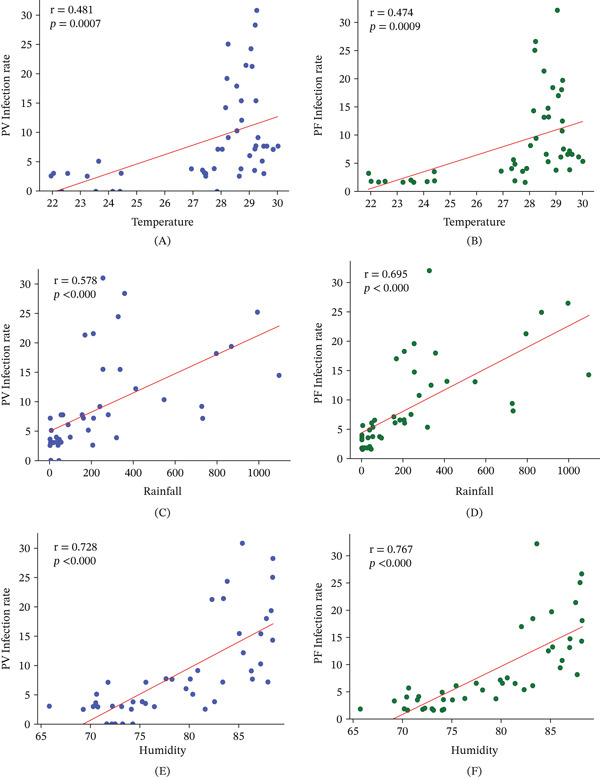
Correlation between meteorological variables and *Plasmodium* infection rates in Cox′s Bazar (2021–2024). (A–F) Scatter plots illustrating the relationship between monthly meteorological parameters and infection rates of *P. vivax* (*Pv*) and *P. falciparum* (*Pf*). (A) Temperature versus *Pv* infection rate (*r* = 0.481, *p* ≤ 0.001), (B) temperature versus *Pf* infection rate (*r* = 0.474, *p* ≤ 0.001), (C) rainfall versus *Pv* infection rate (*r* = 0.59, *p* ≤ 0.001), (D) rainfall versus *Pf* infection rate (*r* = 0.767, *p* ≤ 0.001), (E) humidity versus *Pv* infection rate (*r* = 0.728, *p* ≤ 0.001), and (F) humidity versus *Pf* infection rate (*r* = 0.77, *p* ≤ 0.001).

Pearson correlation analysis confirmed significant positive relationships between meteorological parameters and malaria infection rates (Figure 3). At Lag 0, all meteorological variables demonstrated significant positive correlations with both *P. vivax* and *P. falciparum* infection rates. Relative humidity exhibited the strongest association (*Pv*: *r* = 0.728, *p* ≤ 0.001; *Pf*: *r* = 0.767, *p* ≤ 0.001), followed by rainfall (*Pv*: *r* = 0.578, *p* ≤ 0.001; *Pf*: *r* = 0.695, *p* ≤ 0.001). Temperature showed comparatively moderate correlations (*Pv*: *r* = 0.481, *p* ≤ 0.001; *Pf*: *r* = 0.474, *p* ≤ 0.001). These contemporaneous associations are visually represented in Figure 3.

Lagged analyses revealed (see Table 2) that these relationships persisted, albeit with reduced strength, across subsequent months. At a 1‐month lag, humidity (*Pv*: *r* = 0.638; *Pf*: *r* = 0.647; both *p* ≤ 0.001) and rainfall (*Pv*: *r* = 0.638; *Pf*: *r* = 0.619; both *p* ≤ 0.001) remained strong predictors of infection rates, while temperature showed moderate but significant associations (*Pv*: *r* = 0.404, *p* = 0.005; *Pf*: *r* = 0.425, *p* = 0.003). At a 2‐month lag, correlations weakened further but remained statistically significant for most variables, particularly humidity (*Pf*: *r* = 0.410, *p* = 0.005) and rainfall (*Pf*: *r* = 0.425, *p* = 0.003).

**Table 2:** Lagged correlation analysis between climatic variables and malaria infection rates (*Plasmodium vivax* and *Plasmodium falciparum*) in Cox′s Bazar, Bangladesh (2021–2024).

Variable	Lag (months)	*Pv* (*r*‐value)	*Pv* (*p* value)	*Pf* (*r*‐value)	*Pf* (*p* value)
Temperature	0	0.481	p ≤0.001 ^∗^	0.474	p ≤0.001 ^∗^
Temperature	1	0.404	*p* = 0.005	0.425	*p* = 0.003
Temperature	2	0.372	*p* = 0.011	0.365	*p* = 0.013
Humidity	0	0.728	p ≤0.001 ^∗^	0.767	p ≤0.001 ^∗^
Humidity	1	0.638	p ≤0.001 ^∗^	0.647	p ≤0.001 ^∗^
Humidity	2	0.334	*p* = 0.023	0.410	*p* = 0.004
Rainfall	0	0.578	p ≤0.001 ^∗^	0.695	p ≤0.001 ^∗^
Rainfall	1	0.638	p ≤0.001 ^∗^	0.619	p ≤0.001 ^∗^
Rainfall	2	0.329	*p* = 0.025	0.425	*p* = 0.003

*Note:* “*r*” denotes Pearson′s correlation coefficient, representing the strength and direction of the association between climatic variables and malaria infection rates. *p* values indicate statistical significance of the correlations and were derived using two‐tailed tests. Statistically significant associations (*p* < 0.05) are interpreted as evidence of meaningful relationships. Lag 0 represents same‐month associations, while Lag 1 and Lag 2 correspond to climatic conditions 1 and 2 months prior to observed malaria incidence, respectively. An asterisk (*) indicates highly significant associations (p ≤ 0.001).

### 3.5. Anti‐*Plasmodium* Antibody Dynamics

**Figure 4:**
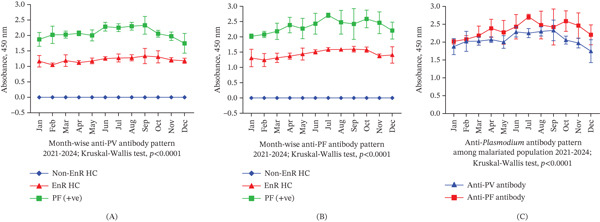
Anti‐*Plasmodium* antibody responses in different cohorts. (A) Mean serum antibody levels across non‐EnR HC, EnR HC, PvAg+, and PfAg+ groups show the highest responses in PfAg+ individuals. (B) Antibody levels consistently elevated in infected groups, especially PfAg+. (C) Comparative analysis reveals stronger humoral responses in PfAg+ cases, with notable differences between endemic and nonendemic controls.

Serological profiling using ELISA revealed markedly elevated anti‐*Plasmodium* antibody levels among individuals from malaria‐endemic regions and those with active infections, with the highest titers observed in PfAg+ individuals, indicating a strong humoral response to acute infection, as illustrated in Figure 4. Antibody levels increased progressively across study groups from non‐EnR HCs to EnR HCs, and further to PvAg+ and PfAg+ individuals reflecting cumulative antigenic exposure. PfAg+ individuals demonstrated significantly higher antibody levels than all other groups (*p* < 0.001), consistent with intense immunological activation characteristics of falciparum malaria. Seasonal analysis revealed peak antibody titers during the monsoon‐driven transmission period (June–September), coinciding with increased mosquito activity. Furthermore, mean monthly antibody levels were positively correlated with ambient temperature, suggesting that climatic factors modulate immune response indirectly through enhanced transmission intensity. These findings highlight the synergistic influence of pathogen exposure and environmental conditions on antibody‐mediated immunity within the Rohingya refugee population.

### 3.6. Hematological and Serological Alterations in Malaria‐Infected Individuals

**Figure 5:**
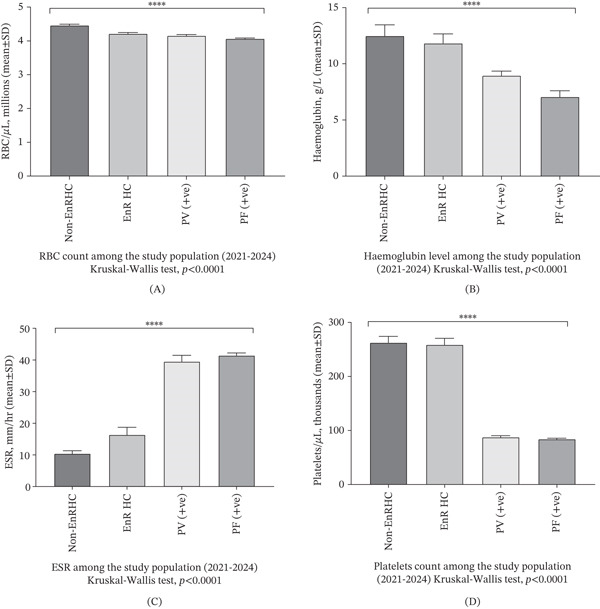
Hematological and serological profiles in healthy and malaria‐infected individuals. Mean values of (A) hemoglobin (grams per liter), (B) RBC count (millions per microliter), (C) ESR (millimeters per hour), and (D) platelet count (cells per microliter) across non‐EnR HC, EnR HC, PvAg+, and PfAg+ groups. Notable decreases in hemoglobin, RBC, and platelet counts, along with elevated ESR, were observed in infected individuals, most prominently in PfAg+ cases. Quadruple asterisk (****) indicates extremely statistically significant differences *p*( < 0.0001).

Hematological profiles revealed significant abnormalities in malaria‐infected individuals compared to healthy controls (Figure 5). Mean RBC counts declined from 4.463 million/*μ*L in non‐EnR HCs to 4.066 million/*μ*L in PfAg+ patients. Hemoglobin levels dropped substantially from 12.50 g/L in controls to 7.055 g/L in PfAg+ individuals, consistent with malaria‐induced anemia. Platelet counts also decreased significantly (84,000–88,000/*μ*L in infected individuals vs. ~260,000/*μ*L in controls), indicating thrombocytopenia. ESR, an inflammatory marker, was markedly elevated in infected individuals (up to 41.43 mm/h in PfAg+), suggesting systemic inflammation. These hematological disruptions were more severe in *P. falciparum* infections, highlighting its greater pathogenicity compared to *P. vivax*.

## 4. Discussion

This study presents a comprehensive analysis of malaria transmission dynamics among the Rohingya refugee population in Cox′s Bazar, Bangladesh, from 2021 to 2024, with a focus on seasonal climatic influences, immunohematological responses, and species‐specific infection patterns. Malaria incidence peaked during the monsoon months (June–September), with July and August consistently reporting the highest caseloads. These findings corroborate previous regional and global studies reporting enhanced malaria transmission during periods of increased rainfall and humidity, which facilitate vector breeding and parasite development [18]. Recent studies conducted in displaced communities in the Democratic Republic of Congo [19] have demonstrated similar seasonal surges, indicating that malaria remains a formidable threat in humanitarian settings where vector control coverage and healthcare infrastructure are often disrupted or inadequate.

The species‐specific analysis revealed that *P. falciparum* infections peaked earlier and more acutely than *P. vivax*, which exhibited a more prolonged and moderate incidence curve. This pattern is biologically plausible given the faster erythrocytic cycle and higher pathogenicity of *P. falciparum*, which also tends to produce more severe clinical symptoms and higher parasite densities [20]. In contrast, *P. vivax* has a latent hepatic stage (hypnozoites), which contributes to relapse infections that may not align with immediate vector exposure [21]. Our finding that *P. vivax* infections often appear slightly offset from the *P. falciparum* peak is supported by recent modeling studies from Southeast Asia [22], where asynchronous transmission cycles have been attributed to the differing biology and relapse mechanisms of these parasites [23].

The statistical analysis demonstrated strong positive correlations between malaria incidence and key climatic variables. Both relative humidity and rainfall showed significant associations with *P. falciparum* and *P. vivax* cases, aligning with contemporary research showing that climatic thresholds modulate vectorial capacity and extrinsic incubation periods [24, 25]. Temperature emerged as a critical determinant, with transmission rising notably when average temperatures ranged between 25°C and 30°C, a range previously identified as optimal for *Plasmodium* development in malaria vectors [26–28]. Our multiple linear regression model, which identified ambient temperature and *P. vivax* incidence as strong predictors of *P. falciparum* transmission (*R* ^2^ = 0.9147), supports the hypothesis that climatic variables can be harnessed for predictive modeling in endemic and epidemic‐prone zones. However, the model′s interpretation must consider other unmeasured cofactors, including population movement, land use change, and control intervention coverage.

The immunological analysis revealed significantly higher titers of anti‐*Plasmodium* antibodies in individuals infected with *P. falciparum* compared to those infected with *P. vivax*. This finding aligns with the known higher immunogenicity of *P. falciparum*, attributed to its complex antigenic structure and capacity to induce more severe clinical symptoms [29, 30]. Notably, antibody levels peaked during the monsoon season (June–September), corresponding with heightened malaria transmission. These elevated titers likely reflect recent or acute infections, while persistently detectable antibodies in asymptomatic individuals suggest cumulative exposure and the development of partial immunity over successive transmission seasons. A recent serosurveillance initiative among forest‐goers in Northeastern Thailand [31] reported similar temporal antibody profiles, emphasizing the role of humoral markers as surveillance tools in high‐risk populations. Interestingly, several participants from the EnR who tested negative by RDT exhibited moderate to high levels of anti‐*Plasmodium* antibodies. This indicates possible subclinical or previously resolved infections, or repeated exposure to infected mosquito bites without progressing to detectable parasitemia. Such findings are consistent with the concept of “semi‐immunity” or “premunition,” where continuous low‐level exposure in endemic areas enhances immunological memory without resulting in clinical illness [32, 33]. It is also possible that these individuals were infected at timepoints not captured by RDT, which detects current parasitemia but not past exposure. In contrast, individuals from the non‐EnR showed no detectable anti‐*Plasmodium* antibodies, supporting the absence or extremely low presence of malaria vectors in that area. This further reinforces the role of sustained vector exposure in generating and maintaining humoral immunity in endemic populations.

Hematological assessments showed common abnormalities in malaria‐positive individuals including anemia, thrombocytopenia, and elevated ESR. These are well‐documented hematological consequences of both *P. falciparum* and *P. vivax* infections, with *P. falciparum* typically causing more severe disruption [34–36]. Thrombocytopenia in particular was markedly higher in *P. falciparum* patients, possibly due to immune‐mediated platelet destruction or splenic sequestration [37]. Anemia in both species may arise from a combination of hemolysis, dyserythropoiesis, and cytokine‐induced marrow suppression, as described in recent meta‐analyses [38]. These hematological changes not only serve diagnostic value but also provide insights into the pathophysiology of malaria in vulnerable populations with potentially compromised nutritional and health status.

Despite these strengths, several limitations should be acknowledged. First, the absence of entomological surveillance limits the ability to directly link vector dynamics with observed transmission patterns. Second, meteorological data were obtained from a single regional station and may not capture microclimatic heterogeneity within Camp 26, thus reflecting regional rather than individual‐level exposure. Third, reliance on RDT with microscopy confirmation, without PCR, may have missed low‐density or subclinical infections. Molecular confirmation using PCR was not performed; therefore, subclinical infections inferred from serological patterns should be interpreted cautiously. Fourth, key variables including intervention coverage (e.g., insecticide‐treated nets and indoor residual spraying), population mobility, and antimalarial resistance were unavailable, introducing potential confounding and limiting comprehensive interpretation of transmission dynamics. Fifth, immunological analysis was restricted to humoral responses without evaluation of cellular or cytokine‐mediated immunity. Additionally, operational constraints, including funding limitations, restricted access to certain camp sectors, and ongoing population influx, may have affected sampling consistency. Finally, while the findings are robust within the refugee context, extrapolation to broader endemic settings may be limited due to the unique demographic and ecological characteristics of this population.

## 5. Conclusion

This study reveals the seasonal and species‐specific heterogeneity of malaria transmission within the Rohingya refugee settlements in Cox′s Bazar and emphasizes the pivotal influence of climatic factors in modulating infection patterns. The observed elevated antibody responses and hematological alterations, particularly in *P. falciparum* infections, point toward both recent and cumulative exposure and suggest a heightened risk of severe clinical manifestations in this vulnerable population. These findings underscore the need for timely, tailored interventions that align with seasonal transmission windows and account for the biological differences among *Plasmodium* species. To strengthen malaria control efforts in such complex humanitarian settings, future research should integrate entomological surveillance, expanded immunological profiling, and drug resistance monitoring. Such approaches will contribute to a more comprehensive, adaptive, and resilient disease management strategy tailored to the unique challenges of displaced populations.

## Author Contributions

A.I. defined the concept. A.I., M.M.A., and P.K.P. designed the study. A.I., M.M.A., and P.K.P. collected samples and meteorological data. A.I., M.M.A., P.K.P., N.R., S.B., and A.H. analyzed the dataset. A.I., M.M.A., P.K.P., N.R., M.R., S.B., and A.H. prepared the manuscript. A.I., S.B., T.M.P., M.R., A.S., M.M.H.C., and M.S.A. edited the manuscript. A.I., M.M.A., S.B., M.M.H.C., and M.S.A. reviewed the manuscript. A.I. and M.M.A. have contributed equally to the study.

## Funding

No funding was received for this manuscript.

## Disclosure

The lead author confirms that the manuscript is an honest, accurate, and transparent account of the study being reported, that no important aspects of the study have been omitted, and that any discrepancies from the study as planned have been explained. All authors have read and approved the final version of the manuscript.

## Ethics Statement

The Mawlana Bhashani Science and Technology University (MBSTU) Ethical and Review Committee authorized the study (MBSTU/ERC/2021/12).

## Consent

Patients or their guardians gave their signed permission after receiving the necessary information. The confidentiality and identity of the patients were protected at all costs.

## Conflicts of Interest

The authors declare no conflicts of interest.

## Data Availability

The data that support the findings of this study are available on request from the corresponding author. The data are not publicly available due to privacy or ethical restrictions.
